# On Collective Molecular Dynamics in Biological Systems: A Review of Our Experimental Observations and Theoretical Modeling

**DOI:** 10.3390/ijms23095145

**Published:** 2022-05-05

**Authors:** Carlo Dal Lin, Paola Romano, Sabino Iliceto, Francesco Tona, Giuseppe Vitiello

**Affiliations:** 1 Department of Cardiac, Thoracic and Vascular Sciences, Padua University Medical School, 35100 Padua, Italy; carlodallin@libero.it (C.D.L.); sabino.iliceto@unipd.it (S.I.); francesco.tona@unipd.it (F.T.); 2 Department of Sciences and Technologies, Sannio University, 82100 Benevento, Italy; 3 CNR-SPIN Salerno, 84084 Salerno, Italy; 4 Department of Physics “E. R. Caianiello”, Salerno University, 84084 Salerno, Italy

**Keywords:** cell responses to sounds, sounds’ effects on cytoskeleton, cellular contractility, fractal and multifractal structures, coherent states, morphogenesis processes

## Abstract

We review processes by which different sounds, such as meditation music, mantra, kindness, or hatred expressions, and noises induce responses from cells and their components. We define ‘good’ or ‘bad’ sounds as those enhancing or inhibiting the cell’s biological activity, respectively. It is highlighted that the cellular dynamics results in a coherent organization with the formation of ordered patterns due to long-range correlations among the system constituents. Due to coherence, in the framework of quantum field theory, extended domains become independent of quantum fluctuations. Non-dissipative energy transfer on macromolecule chains is briefly discussed. Observed fractal features are analyzed by the fast Fourier transform and a linear relationship between logarithms of conjugate variables is observed. The fractal relation to the generation of forms (morphogenesis) and to the transition from form to form (metamorphosis) is commented. The review is also motivated by the suggestions coming from the cells’ responses, which show their ability to move from the syntactic level of the sound component frequencies to the semantic level of their collective envelope. The process by which sounds are selected to be good or bad sounds sheds some light on the problem of the construction of languages.

## 1. Introduction

It has been proposed that sounds (pressure waves) may influence and modulate several biological functions, such as blood pressure, heart rate, respiration, body temperature, as well as cardiac and neurological functions [1,2]. From a cellular level up to a systemic framework, rhythms can produce relevant bioeffects [1]. Every signaling event is modeled by rhythms, and the synchronization of coupled oscillators and dynamical systems is a crucial issue in essential processes of life. There is growing evidence that mechanical vibration deeply affects stem cell dynamics and organ function, and the sounds with their pressure waves may affect cells’ behavior determining micro-vibrations as well as resonances [1]. It is well established that a wide variety of biological processes are influenced by the nanomechanical properties of subcellular structures [3,4,5,6,7]. An example of this is given by the vibrational modes generated by the cytoskeleton and nucleoskeleton, whose resonance patterning can be transmitted to and recorded from the cell surface [3]. The cytoskeleton also plays an important role in defining the mechanical and functional features of cells, regulating transport and governing many cellular processes, such as mitosis and meiosis [1].

**Figure 1:**
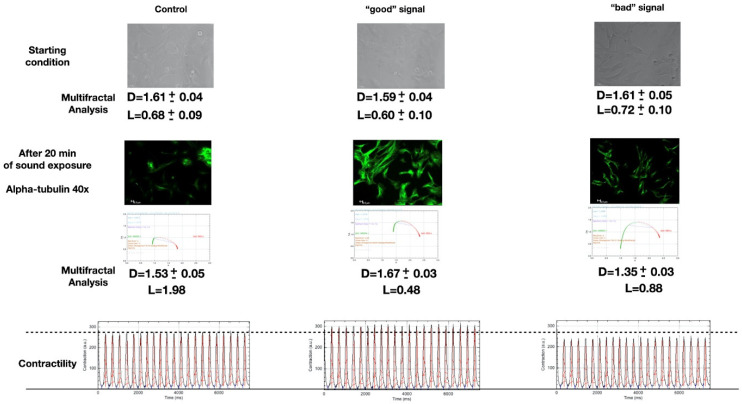
(Modified from [8]). The first row shows the starting condition using bright-field images (representative images selected from 5 positions and 6 experimental repetitions). The cells have a basal multifractal arrangement characterized by an average fractal size (D) of about 1.6 and a lacunarity (L) of 0.7. Scale: 10 μm. The middle row represents the result of the different sound stimulations. Alpha-tubulin was marked in green. In the control cells, there were no significant variations in the parameters D and L. In the case of “good” sounds, there was an increase in the D to 1.7 and a decrease in the L to 0.4. In the case of “bad” signals, the D reduced to 1.3 with a slight increase in the L to 0.8. Scale: 10 μm. The lower row shows the contractility analysis of the same cell under the different acoustic stimulations.

In this review, we discuss the results of our previous proof-of-concept work about the effects induced by different sounds on cells and their components [8]. In Figure 1, a synthesis of our experiment is depicted [8].

In summary, cells of murine atrial cardiomyocytes (HL1) embedded in a liquid culture medium were exposed to 20 min sound sequences (Figure 1, first row), were stained for cytoskeletal markers (such as alpha-tubulin, Figure 1, middle row), and studied with multifractal analysis to monitor in vitro eventual reactions and changes. A single cell was live-imaged and its dynamic contractility changes in response to each different sound were analyzed using Musclemotion for ImageJ (Figure 1, third row at the bottom). Six replicas of the experiment were performed, documenting each time how different sound stimuli influence the contractility and the spatial organization of HL1 cells, resulting in a different localization and fluorescence emission of cytoskeletal proteins.

Processes may occur by which the energy supplied by ‘good’ sounds to the biological matter is used for the creation of order, formation of protein ‘bridges’, cell-to-cell correlations, strengthening of the links in the cytoskeleton network, etc., resulting in enhanced biological activity. The ‘bad’ sounds instead induce a negative response, i.e., the inhibition of the ordering among biological components. Fractal behavior plays a relevant role in the contractility measurements performed on cardiac muscle cells using Musclemotion for Image [8].

Acoustic stimuli have been thus demonstrated to influence the individual and collective behavior of cells.

A theoretical analysis has been presented [8] to account for the main aspects of the phenomenology of the interaction between sound waves and cells. Vibrational modes of the electric dipoles characterizing the cellular molecules are considered to be quantum variables and quantum field theory (QFT) formalism is needed to properly describe them. The role of the phonons (i.e., the quanta of the sound waves) has been considered, providing a deeper understanding of the interaction of sound waves with cells. Moreover, the theoretical modeling can account for the experimental observation of the fractal and multifractal self-similarity [9] in the response signals of cells to sound stimuli. An isomorphism has been found between the coherent state molecular regime and fractal and multifractal structures [10].

Several specific mechanisms can be invoked, such as phase locking and phase content in the dynamics of the molecular components and the water bath in which molecules and cells are embedded [11,12,13,14,15]. Additionally, non-trivial topological properties may have an important role in the molecular dynamical properties [14,15,16,17]. Our review will include an illustration of the QFT spontaneous breakdown of symmetry (SBS), a mechanism that is the basis of most theoretical and experimental physics. Ordered patterns and organization appear as a consequence of SBS [18,19,20,21,22].

The motivation for this review and discussion is the need to relate among themselves the two main approaches by which biological systems are usually investigated. In the first, most common approach, the many elementary components of the living systems are studied separately from each other. The second approach is based on a systemic view of understanding biological activity. Collective dynamic modes and synchronization of spatially separated molecular subunits are supposed to play a key role in understanding how to describe the overall behavior of a large number of molecular components. The highly ordered pattern in the spatial molecular arrangements indicates that the study of the biochemical properties of interacting molecules needs to be complemented by the study of their dynamics in the framework of QFT. This indeed accounts for the collective molecular long-range correlations, which are responsible for the observed organization, regularities, and high functional efficiency in the behavior of biological systems, out of reach from the study limited to the properties of each component. This last one is, of course, essential; it is necessary, although not sufficient, to the understanding of the system’s functional activity [8,14,15].

One further motivation in our review is to analyze the role played by the formation of ordered patterns (fractal *forms*) consequent to the process of SBS in terms of *morphogenesis* and *metamorphosis* processes (see Section 3 and Section 4) [8,23].

Finally, the fact that the experiment cells give the same response to vocal sounds pronounced by different persons suggests interesting views on the language, also concerning results from subjects experiencing the relaxation response (RR) practice analyzed in Ref. [24], which we briefly mention in the following (cf. Section 2 and Section 3). Among other consequences, such an RR practice has induced also changes in the language used by the patients undergoing a specific protocol of RR sessions. For example, a statistically significant decrease has been recorded [24] in the use of first-person singular pronouns, while an increase has been recorded for the first-person plural pronouns, with a decrease in the total, singular plus plural, first-person pronouns; a decrease in the use of negations and articles in the sentences; an increase in positive expressions of affective and emotional processes, etc. These changes in the language, at a high level of organization complexity, have been accompanied by variations in biological markers, serum transparency, and pH, at the biochemical, say microscopic, level [24]. The question thus arises of how these two levels, each one with its complex, although different, dynamical features, are correlated. Of course, we do not mean that the language level, involving also the extremely complex brain functional activity, might be naively, in an oversimplified way, traced back or described in terms of the observed cell interactions with sounds, which are reviewed in the following. On the contrary, our discussion only provides a possible first step toward the understanding of how highly complex functional activities, such as the ones related to the language, might be interrelated with the biochemical background activity.

## 2. Results Summary

The results of our previous proof-of-concept study show different responses from HL1 cells exposed to 20 min sound sequences of different sounds, without a regular pattern of high and low frequencies and rhythms from vocal sounds, kindness or hatred expressions, to mantra, meditation music, or noises. One of the motivations to expose cardiac HL1 cells to the specific time of 20 min was the study in vitro of the effects of sounds to understand the results obtained in vivo with patients following Relaxation Response sessions of 20 min each, listening to music, words, and other sounds which, as said at the end of the previous section, turned out to affect their heart functions and other biological markers (e.g., blood serum transparency, pH, etc.) [24,25,26]. The choice of atrial cardiomyocytes was suggested by their specific properties of contractility and of forming networks, which permits visualization of contraction and conformational changes as responses to the stress induced by the sound waves. The technique used for such observations was the time-lapse technique (see Ref. [8]).

The graphs shown below each image in the first and middle row of Figure 1 represent ƒ(α) vs. α, called the multifractal spectrum, i.e., the typical pattern in the fractal analysis [9,27]. *f*(α) can be viewed as the fractal dimension of the set formed by sub-sets with local scaling exponent α. The relation of *f*(α) and α with the generalized dimension D is through a Legendre transformation [27,28]. All ƒ(α) curves form humps over a broad area, as expected for multifractal behavior. The amplitude of the resulting curve (with the shape of a parabola) reflects the degree of irregularity in the distribution of the points. The lacunarity (L) gives a measurement of such inhomogeneities. The parabola degenerates to a point in the case of a (mono-)fractal. The uniform distribution of points is for α = 1, while α < 1 (and α > 1) represents a distribution of points ‘dense inside and dispersive outside’ (and ‘dispersive inside and dense outside’). The resulting average fractal dimension D together with L is also indicated for each case.

**Figure 2:**
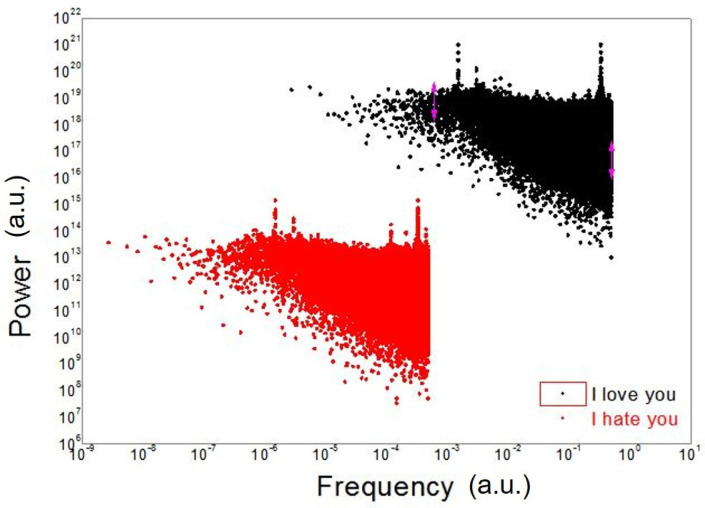
FFT of the “I love you (ti amo)” (black points, upper graph) and “I hate you (ti odio)” (red points, lower graph) signals used in our previous work [8].

The fast Fourier transform (FFT) analysis has also been performed for each signal, showing interesting results. In Figure 2 the FFT, performed with OriginLab software, is reported for two of some of the sounds used to stimulate the cells, in particular, the (good) signal “I love you” and the (bad) signal “I hate you”.

Although it is not possible to distinguish a regular linear behavior in these signals, the analysis performed in [8] showed a multifractal nature in both cases, where a multifractal structure can be described as made of fractal sub-structures. However, while the “I love you” signal seems to favor a very homogeneous multifractal arrangement in the cells (parameter D), the “I hate you” signal presents very inhomogeneous values of the D parameter.

In biological matter, macromolecules are characterized by the specific electric dipole moment of their constituent units. Macromolecules and cells are embedded in the water bath, and water molecules are as well characterized by their specific electric dipole moment. Since dipoles may be oriented in any direction, the basic symmetry is the rotational spherical symmetry. The action of sounds may induce a breakdown of this dipole symmetry by producing a non-vanishing polarization density *P*(*x*,*t*) (dipole ordering). The dipole long-range correlations arising in such a process of symmetry breakdown generate the collective, coherent behavior of the system components, which manifests in the macroscopic scale behavior of the observed cellular structures and cell network. The ordering is strictly related to fractal self-similarity [10]. Self-similarity can be observed through the analysis of the spectrum on a log–log scale since it unveils both periodic structures and scaling laws.

**Figure 3:**
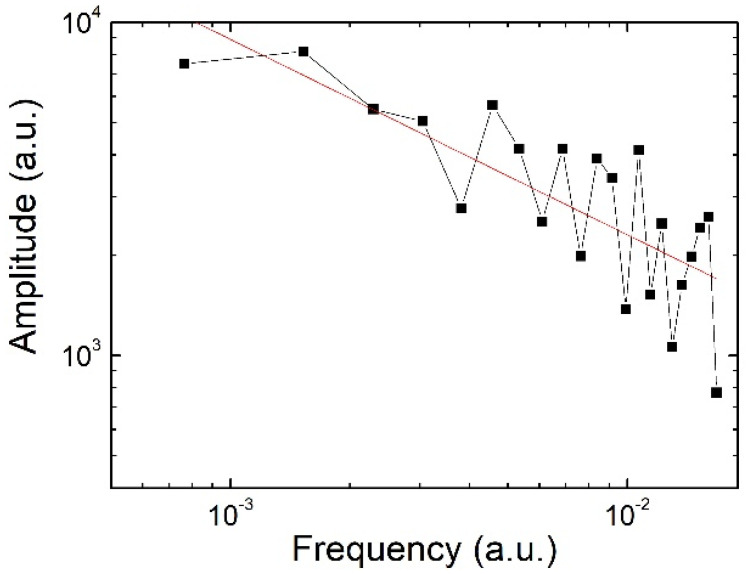
Amplitude of the mantra signal FFT (modified from [8]). Black square denote measurements.

The amplitude of the “Mantra” signal FFT reported in Figure 3 shows for instance a first power-law (scaling law) behavior. Because of the isomorphism between self-similar fractal structures and coherent states, the log–log plot shows indeed that a dynamical coherent regime exists, related to long-range correlation modes at microscopic levels. Only the “Mantra” signal shows a monofractal behavior, as also confirmed by the immunofluorescent signal emission plotted as a function of frequency on a double logarithmic scale (see Ref. [8]).

As mentioned, such a fractal-like self-similar structure is the manifestation of coherent dynamics active at a microscopic level.

Water constitutes the environment in which macromolecules, all the cell components, and cells themselves are embedded. The radiative interaction (not the static one) between the cell components and water, as well as the one with enzyme macromolecules, is then considered [8,14,15]. The collective dipole behavior has been studied in the framework of QFT, according to which any interaction between two systems is mediated by the propagation of a field or quanta, such as, for example, photons exchanged between interacting electric charges in quantum electrodynamics.

All the graphs are expressed in arbitrary units (a.u), commonly used in physiology or spectroscopy, as a relative unit of measurement to show the ratio of the intensity of the signals analyzed or of the pixel displacement—as an estimate of a “contractility” trend and “spectral intensity” trend—to a predetermined reference measurement [28].

In summary, the a.u only serves to compare multiple measurements performed in similar environments, since the ratio between the measurement and the reference is a consistent and dimensionless quantity independent of what actual units are used [28,29].

## 3. Discussion

HL-1 cells’ responses to sound are different for different sounds. The sounds used in the experiment are made by a spectrum of frequencies that, propagating in the liquid culture medium, seem to stimulate cellular organization. This biological reorganization exhibits the fractal or multifractal self-similar spectral structure, as shown by the log–log plots in Figure 1 (third row), Figure 2 and Figure 3, unveiling periodic and scaling laws.

The effect of sound seems thus to be connected to its specific fractal geometric form, directly modulating the cytoskeleton morphology through penetrating up to the nucleus of cellular microvesicles [30] and influencing protein oscillatory motions in the cytoplasm. At the same time, electromagnetic inter-molecular interaction drives the fundamental processes for the cell survival [14,15,30,31]. Since the protein chains may support wave excitations as well as nonlinear solitary waves [8,14,15,16,17], sounds and proteins may undergo an interaction by exchanging quantum modes, e.g., dipole quantum waves, through the mediation of the liquid bath in which they are embedded. Oscillatory and assembly modes will then be present according to the sound characteristics [32,33].

The studied cellular contractility patterns represent different cell responses, in some cases (“good signals”) presenting an increase in contractility while in other cases (“bad signals”) decreasing it. This seems to concur with other results on the response of human cells to “good sounds” (such as melodic music and human voice) measured through a multi-spectral imaging system.

The observed reaction of the cells to sounds, reported in the previous section, may cooperate with the possible biophysical and molecular mechanisms underlying the heart–brain correlation discussed by some of the authors (CDL and GV) in Ref. [34]. There, they have shown (see also [24]) how the practice of relaxation response (RR) improves several biological functions, for instance coronary blood flow, inducing positive changes in molecular inflammation processes, stress hormones, neurotransmitters, aging markers, and circulating microRNAs. Furthermore, the serum changes during RR [8,24,25], and even language, may change with respect to the complex perceptual experiences felt by the body. The information contained in the sounds and phonemes used during every 20 min RR session seems thus to directly influence bodily functions and the heart, in particular, responses that seem to be indeed consistent, at a cellular and molecular level, with the effects of sounds on cells reported above.

At the level of theoretical modeling, the experimentally observed responses of cells to different kinds of sound stimulations can be understood by realizing that, through a chain of steps, “good” (or “bad”) sounds induce (or contrast or destroy) the formation of long-range correlations among the system elementary constituents. These long-range correlations describe the ordering induced by the mechanism of spontaneous breakdown of symmetry (SBS). SBS consists of the fact that the ground state of the system, i.e., the lowest energy state, is characterized by symmetry properties that are not the same as the motion equations defining the system dynamics.

The symmetry of the equations is then said to be spontaneously broken (‘spontaneously’ since the symmetry of the ground state is ‘dynamically’ singled out). The system’s elementary components are the electric dipoles of the water molecules inside the cells and of the bath in which the cells are embedded. They constitute about 90% of the present molecules of the studied system. In the initial step, the input sounds induce, as mentioned, the breakdown of the dipole spherical symmetry by producing, if a ‘good’ sound (or contrasting, if a ‘bad’ sound), the dipole polarization density *P*(*x*,*t*). The dynamical effect following from the SBS is the formation of long-range dipole correlations resulting in the “in-phase” *collective* motion of the dipoles (ordering). The value of *P*(*x*,*t*) gives a measure of the degree of ordering induced by SBS and is indeed called the order parameter [14,15]. It is a classical field, independent of quantum fluctuations.

The quanta associated with the long-range correlations, called Nambu–Goldstone (NG) quanta, have zero mass and integer spin (are bosons), which means that a large number of them may *condense* in the ground state of the system, forming a *coherent* state. ‘Coherent’ means that long-range correlations coexist in that state without negative interferences. They are long-range “phase correlations” among oscillating molecular dipoles.

We remark that the dipole long-range correlations reduce the randomness of the molecular kinematics, facilitating short-range interactions (Van der Waals interactions, H-bonding, etc.), thus allowing the observed high efficiency of biological systems, otherwise incompatible with purely random chemical activity.

We also observe that an electromagnetic (em) field (of external or internal origin) is not able to propagate within the correlated region if its strength is too weak. If it is too strong, it may destroy the correlations. For an intermediate strength, the field can percolate the coherent region by propagating in a filamentary or self-focusing way [15,35,36,37,38,39] (similar to self-focusing in the Kerr effect in nonlinear optics or to the Anderson–Higgs–Kibble mechanism [40,41,42] in QFT). The condensation persists outside the propagation channel; it is zero inside the channel. By using such a mechanism, a model for the formation of microtubules within the cell aqueous environment has been formulated [15] and the internal diameter of the microtubules has been computed to be about 146 Å, fitting quite well the experimental value of about 150 Å (see Appendix A.1).

This process contributes to the understanding of the observed dynamical rearrangement, formation, and depletion of the cytoskeleton microtubules under the stimuli of good and bad sounds, respectively. The cell functional activity may thus occur within a limited window of high and low values of the ordering produced by external stimuli (thus defining the limits and the meaning of “good” and “bad” sounds).

In addition to the SBS phenomenon, sounds may produce at the extremity of a quasi-unidimensional structure, e.g., a protein chain, a molecular deformation turning into oscillations of the electric dipoles of the chain units. The molecular deformation (space displacement field) may couple to the molecular dipoles and produce a wave by which the localized deformation propagates over the chain according to the dynamics of the nonlinear Schrödinger equation. Such a wave is the so-called Davydov solitary wave or Davydov soliton. The energy carried by this wave propagates in a non-dissipative way since the soliton remains stable when colliding with obstructions or other waves [14,43,44,45]. The relevance of such a phenomenon to the energy supply, transport, and storage in biological systems is obvious (cf. Appendix A.3) [14,15,43].

The energy supplied in various forms to the cells, thus also by “good” (“bad”) sounds, can be generally regarded as the “feeding” of the cells, provided of course the supplied energy does not produce thermalization of (heating up) the cell and its environment, destroying dipole ordering (“bad” sounds). The resulting electret’s state (the dipole in-phase ordered patterns), in the “good” case, has a short lifetime, so cyclic feeding processes are necessary to keep efficiently active the cellular functional properties.

Fractal self-similarity has been also found in experimental studies and analysis of DNA and coherent long-range molecular organization has been suggested [46,47].

The mentioned isomorphism between fractal self-similarity and coherent states suggests that the experimentally observed fractal structures reported in the previous section are manifestations of the coherent dynamics of the long-range dipole correlations. Such processes of ordering are processes generating *forms* with fractal properties. It is then intriguing to think of them as *morphogenesis* processes [10]. This might shed some light indeed on the morphogenesis dynamics, a problem (at least) not completely solved in biomolecular studies. Moreover, along a similar line of thought, we are also motivated to study the transitions through different ordered states (*phase transitions*) as processes from *form* to *form* and think of them as *metamorphosis* processes [14,15,23], so recurrent in the *life*, i.e., the time evolution, of biological systems.

Finally, the experimental observation that cells react with the same responses to different persons’ vocal sounds suggests that cells’ response is induced not by individual frequencies, voiceprint, and tones, but by their *collective* envelope. In this way, cells perform very fast and efficient transitions from the *syntactic* unit’s level (individual frequencies) to the *semantic* level, a not easy task to be achieved by an ‘artificial’ device. Such behavior might contribute, at the cellular level, to the observed changes in the languages used by patients after relaxation response (RR) practice [24], as mentioned in Section 1. Such an RR practice has been documented [25,26] to produce in the subjects not only positive effects in the inflammation molecules, stress hormones, neurotransmitters, and aging markers, including serum transparency, but also changes in the words and structures of language expressions [24] denoting changes in the meaningfulness of the subject cognitive experiences and emotions.

The RR practice thus leads to the intriguing result of relating the microscopic complex biochemistry at a cellular level to the macroscopic complex functional level of language use, with lexical choices and sentence construction. Such a transition from micro-to-macro and vice versa is of course far from being a trivially linear process of cause-to-effect. It denotes instead a highly non-linear process by which *collective modes* (i.e., *behaviors* at the cellular level and functional macroscopic level) are dynamically generated, both ways (down-up and also up-down), given the observable *responses* at the biochemical level that the *relaxation* practice produces. This might shed some light on millenary wisdom about the *powerfulness* of “a good word” or of “a bad word”.

There is a further suggestion coming from what is observed to be “good” and “bad” sounds, for example in the kindness or hatred expressions. Of course, the cell does not know the meanings of the words. The suggestion is that an “opposite” mechanism is at work, in the sense that, at the level of macroscopic behaviors, our ancestors had the experience that “certain” sounds were producing “good” effects on the receiver, while other sounds were producing opposite, “bad” effects on him/her. Then, those specific sounds producing a “good” response were adopted in the language as expressions of kindness, and similarly for the bad ones as expressions of hatred and hostility. Originally, the language has been thus constructed based on the reaction of the receiver. In this way, people “learned” that some words were producing good effects on the receiving subject, and therefore they “adopted” those words in their language as kindness words. As a matter of fact, this describes how we “learn to talk” with our pets. Their reactions to our words, or sounds in general, “teach” us how to talk to them. We learn from them how to speak to them. It is not that they learn our language. These remarks deserve further study, which we plan to do in the future, also considering some fractal structures emerging in the language [48].

We finally observe that the analysis in terms of em frequencies done by Geesnik and Meijer in Ref. [49] could be also applied to the spectrum with fractal–multifractal self-similarity manifesting in the log–log plots of Figure 1, Figure 2 and Figure 3.

In summary, the picture emerging from the theoretical modeling seems to fit well with the experimentally observed effects induced by ‘good’ and ‘bad’ sounds.

## 4. Conclusions

The experimental observation shows that the energy supplied by sounds to the cells could produce ordering, protein ‘bridges’, formation and strengthening of links in the cytoskeleton network, etc., thus stimulating the biological activity. Such sounds have been classified as “good” sounds. The “bad” sounds induce, on the contrary, inhibition of ordering among biological components, even the cell explosion (see Ref. [8]).

Observations have also shown the recurrence of fractal and multifractal structures in the cell response to sound stimuli, which represents a characteristic feature of these experiments, and signals that the underlying dynamics is a coherent dynamics, according to the theorem that an isomorphism exists in QFT between coherent states and fractal structures [10]. In the adopted theoretical modeling, good sound promotes the formation of coherent long-range correlations among the electric dipoles of the water molecules of the bath in which cells and their components are embedded. Bad sounds, instead, oppose the formation of these long-range correlations or destroy them if they already exist. In this view, remarkably, the theoretical modeling can describe the formation of microtubules in terms of a process of molecular coating of channels produced in the coherent ordering by em fields of external or endogenous origin. The internal diameter of the microtubules, computed in such a scheme, turns out [15] to be about 14.6 nm which agrees quite well with the measured diameter of about 15 nm (cf. Appendix A.1).

In connection with the formation of coherent structures, we mention that in quantum mechanics (QM) it is known that the phenomenon of decoherence occurs. However, our theoretical modeling is framed in QFT where coherence represents a quite stable phenomenon [50], for example in crystals, ferromagnets, superconductors, and in a wide range of temperatures, e.g., the coherent ordering of the diamond crystal is lost at the critical temperature of 3545 °C at atmospheric pressure, sodium chloride (the kitchen salt) crystal melts at 804 °C, while the critical temperature for superconductors containing compounds of niobium is −252 °C. The microscopic dynamics of these long-lived ordered systems, persisting even at a high temperature, is indeed ruled by QFT, not by QM. In our theoretical modeling, we thus exploit the fully QFT phenomenon of the spontaneous breakdown of symmetry (SBS), generating, according to the Goldstone theorem [18,19,20,21,22,51], the long-range correlation quanta, the Nambu–Goldstone (NG) quanta, that coherently condense in the system ground state, and manifest in ordered patterns (*forms*). Examples of NG quanta are the magnons, i.e., the spin-wave quanta in ferromagnets; the phonons or elastic wave quanta in crystals; and the ‘dipole wave quanta’ in the present case, associated with the long-range correlations among the electric dipoles of the water molecules.

Biological systems live at multiple organization levels. The interaction of cells with the sound stimuli manifests in the continuous rearrangement of the microscopic equilibrium of the long-range correlations among the basic constituents, thus pursuing the adequate response of the system to the environment’s action on it. The equilibrium is reached by continuously regaining the minimization of free energy (cf. Appendix A.2), re-establishing the balance between the energy supplied by sounds and the formation/rearrangements of ordered patterns (*morphogenesis*) [10]. In such an evolving (*living*) through different dynamical regimes (*phase transitions*) toward a further, although temporary, dynamical equilibrium, from *form* to *form*, we recognize the dynamics of *metamorphosis* processes [10,23] characterizing the continuous activity of the biological system.

We have mentioned above that, in the experiment, vocal sounds pronounced by different persons stimulate the same response. This might suggest that cells in their dynamic reactions to sounds exhibit a sort of quantum computing architecture simulating computational processes. As already remarked, obtaining the *same* response by the cells to different *voices* pronouncing the same words or sequences of words signals that cells can go from the *syntactic* level of individual frequencies to the *semantic* level, detecting the collective frequency modes (meaning) which is what effectively determines the cell response. It is the ‘meaningfulness’ of the RR experience to the specific patient, operating at his profound biological levels, that affects not only biochemical markers but also his language structures and expressions [24,52]. These interesting features certainly deserve further studies that we plan to do in future work.

In this line of thought, we have observed in previous sections that the cell responses induce us to classify the sounds as “good” ones or “bad” ones, according to the different effects by them caused on the cells. This leads us to consider, from such a perspective, the intriguing problem of the ‘construction’ of languages at the dawn of the human communities’ evolution. Perhaps it has been the reaction of the ‘receiver’ to induce the ‘speaker’ to choose a specific ‘sound’ to enhance, damp, or avoid that reaction, something like we do when talking with our pets, trying to understand how to get their attention or how to induce them to assume or not assume a specific behavior. Remarkably, as in the experiments here reviewed, linguistic studies show that fractal self-similarity plays an important role also in language structuring and sentence formation [24,48].

## Data Availability

Not applicable.

## Appendix A

In this Appendix, we review some aspects of QFT and SBS, referring in particular to the observations presented in the text showing that sounds produce conformational changes in the cytoskeletal structures. We therefore briefly review the dynamics underlying the microtubule formation and the temperature relation with the range of the correlations among the system components (the dipoles of water molecules). Energy transfer on linear structures by solitary waves is also reviewed.

### Appendix A.1. Formation of Microtubules

As mentioned in Section 3, an electromagnetic (em) field of convenient strength propagates through coherently ordered regions in a filamentary-like or self-focusing fashion [15,35,36,37,38,39]. One can show that the em field acquires then a mass *M* which depends on the medium polarization density *P*(*x*,*t*), *M = M*(*P*), where *P* denotes *P*(*x*,*t*), and *M*(*P*) *=* 0 at *P =* 0 [15]. One can also show that the diameter of the ‘channel’ through which the em field percolates the coherent medium is *d* = ℏ/*cM*, for *M* ≠ 0, *P* ≠ 0. Here, ℏ=h/2π, with *h* being the Planck constant and *c* the speed of light. Assuming that all the dipoles are correlated, one obtains *d* = 146 Å with *M* =13.60 eV, to be compared with the observed diameter of microtubules ranging from 120 to 150 Å. When *P*(*x*,*t*) comes from about 70% of dipoles, the value *d* = 125 Å is obtained, still in agreement with observations.

Within such channels the em field is non-zero. Outside them is zero. There is, therefore, perpendicular to the channel, a transverse field gradient force *F*(*ν*), *ν* is the field frequency, attracting or repelling, according to a resonant pattern $\;(\nu_{0k\;}^{2}-\nu^{2})$ [15], atoms, ions, molecules of specific frequency $\nu_{0k}$, floating outside the channel. Note that small changes in the frequencies may produce switching between the ± signs in the difference $\;(\nu_{0k\;}^{2}-\nu^{2})$ (thus between attractive/repulsive actions). Note that the field frequency may be influenced by the molecules previously attracted (or repelled) and in general by the molecular environment. The effect is the attraction (or repulsion) of different molecules on the channel boundary, and thus a selective polymerization process leading to the molecular coating of the channel, namely the dynamic formation of the microtubule.

If the em field in the channel disappears, the coating disassembles or persists, depending on the lower or higher chemical affinity of the coating molecules. Moreover, the em field produces also a longitudinal force [15] along the channel, pushing the molecules, thus favoring their chemical interaction, or, depending on their chemical affinity, producing the disruption of the microtubule (especially in its ending part, as, e.g., in the microtubule treadmilling). As well known, most of the metabolic activity occurs on the cytoskeletal microtubules. These also offer a “chemical transport rail” for the cell. The attractive or repulsive transverse em force may then contribute to the interlocked chemical reactions on the microtubule (their sequential ordering), while the longitudinal em force contributes to the chemical transport in the cell.

The NG dipole wave quanta energy *E =* 13.60 eV, corresponding, as seen, to the microtubule diameter of about 146 Å, is the hydrogen ionization energy. It acts as threshold energy. The propagation of the em field (a photon) of energy hν > 13.60 eV may destroy the coherent dipole ordering, and thus restore the spherical wave (Maxwell) em propagation with consequent inhibition or destruction of cytoskeletal microtubules and also induce possible ionization events. A photon of lower energy, instead, cannot penetrate the dipole ordered patterns. In this case, the supplied energy may be ‘stored’ in the system by contributing to the polarization, until the accumulated amount is enough to be used by some chemical reaction of the metabolic activity, or, at the threshold of 13.60 eV, in some ionization processes, or to open a channel through which to propagate (delayed propagation and radiation) [15,26]. Coherence thus offers the possibility to store ‘non-thermalizing’ energy in the system.

In conclusion, the processes of microtubule formation sketched above may contribute to the understanding of the observed dynamical formation of cytoskeletal networks, cell-to-cell correlations, cell mobility, and contractility under sound stimuli, with dependence on their frequency patterns and intensity.

### Appendix A.2. Temperature and Correlation Range

Long-range correlations coexist without negative interferences in the coherent state. Consider then the approximation that the associated NG quanta behave as free particles and also that their mass gets non-zero contributions from boundary effects. Then, we have [15] *p*^2^/2*m_eff_* = (3/2)*k_B_ T*, where *p* is the momentum, *T* the temperature, *k_B_* the Boltzmann constant, and *m_eff_* the effective mass of the NG quantum due to the finite size *R* = ℏ/*c m_eff_* of the coherent domain. By using *R* = *n λ*/2, with *n* an integer and *λ* the de Broglie wavelength *p* = *h*/*λ*, we obtain *R* = *n*^2^*πhc*/(6*k_B_ T*). For *n* = 1 and *T* = 300 K, *R* = 25 µm, consistently with the observed size scale (cf. the scale in Figure 1). Suppose that *T* increases (remaining, however, below the destructive threshold) due to, e.g., an external supply of energy. The system then reacts, to maintain *T* constant, by increasing the size *R* of the correlated coherent domain (for fixed *n*). The range over which coherent correlations span plays thus an important role in controlling the system temperature within a certain functional interval, which of course confirms the utmost relevance of temperature in the living matter [53].

The stationary state condition ((almost) constant *T*, at a given condensation density *N* of NG quanta), is obtained by minimization of the free energy *F*: *dF* = *dU* − $k_{B}$*T dS* = 0, *U* is the internal energy, and *S* is the entropy. This implies [13,51]: $dU\propto \;dN/dt\;\propto k_{B}TdS$ (with heat given by *dQ* = $k_{B}$*T dS*). Thus, a change *dU* > 0, due, e.g., to a supply of energy, implies *dS >* 0 and an increase in the rate of change of dN/dt, i.e., an increase in the loss of correlations in the ground state (*dS >* 0 means indeed loss of ordering). This may explain how ‘disordering effects’, due to ‘bad’ sound supplying energy in a ‘non-resonant’ way to the system, may lead to the observed cell explosion (*dU* > 0); ‘good’ sounds produce, on the contrary, ordering, with *dS* < 0, i.e., *dU* < 0 and a reduction in the loss of correlations, *dN/dt* <0. Energy dissipation (*dU* < 0) (below a critical threshold) plays a positive role in keeping the health state of the system.

In conclusion, variations in *T* turn into variations in the entropy *S*, namely ordering/disordering, through variations in the size *R* of the correlated domains, and vice versa. In these processes, part of the internal energy is ‘used’ to create correlations (*R* increases) or, on the contrary, energy ‘stored’ in the correlations is released to produce increases in the kinetic energy, thus in *T* (in the internal energy *U*). These exchanges of energy between *U* and *S*, *U ↔ S*, constitute a ‘dynamical degree of freedom’ through which the system microscopic configurations may be rearranged, within the equilibrium constraint *dF* = 0 [54].

A final remark is that, due to dynamical nonlinearity, the coupling of the quanta of sound waves (the phonons) to the em field of molecular dipoles is enhanced by √*N*, with *N* the number of components [14,15]. The collective interaction, for large *N*, has then a time scale shorter by a factor 1/√*N* than the short-range interaction time scale among the individual components and is thus not affected by thermal fluctuations for *k_B_ T* less than the energy of the collective interaction.

### Appendix A.3. Solitary Waves

Sounds may couple directly to macromolecule chains (e.g., proteins), or, as mentioned in Section 3, they may trigger ATP reactions, which in turn induce the formation of solitary waves (solitons) on the chain. It is indeed remarkable that the energy to excite the Davydov soliton, of the order of 0.25 eV, is about the same as the energy released by ATP hydrolysis at the origin of a protein chain [14,43,44]. In both cases, the energy released by sounds propagates [14,43,44,45] on the chain, “trapped” in the soliton coherent localized deformation, over large distances due to the soliton (almost) non-dissipative dynamics, and with a time-dependent velocity due to the discreteness of the chain and the consequent periodic potentials [44].

The em radiation produced by the coherent oscillations of the chain molecular dipoles, within the traveling solitary wave packet, breaks the rotational symmetry of the surrounding water molecular dipoles, contributing to the formation of long-range ordering correlations. Moreover, the soliton may capture and trap an electric charge in its motion (electrosoliton) [43,44,45]. The radiation emitted by the non-dissipative electric current flowing (with the soliton) over the chain, together with the radiation from the chain dipole oscillations, may synchronize with the radiation emitted by solitons on the other chains, traveling at the same velocity and emitting radiation at the same frequency [45].

The lifetime of the soliton on the chain is finite since the chain has a finite length. The energy released when the soliton decays propagates in the water bath, not in a diffusive way, but in waveform since the electret state has been previously formed in the bath by the same soliton. The reciprocal dynamical dependence between the molecular chain deformations and the water environment has been observed by spectroscopic experiments [55], where the hydration water forming, under ambient conditions, a coating superstructure of macromolecules (DNA, in Ref. [55]) has been studied.
